# Sampling Quantum States with Inequality Constraints

**DOI:** 10.3390/e28060614

**Published:** 2026-05-29

**Authors:** Weijun Li, Rui Han, Jiangwei Shang, Hui Khoon Ng, Berthold-Georg Englert

**Affiliations:** 1 Department of Physics, University of Oxford, Oxford OX1 3RH, UK; 2 BiQut, Singapore 288564, Singapore; han.rui@quantumlah.org (R.H.); 3 School of Physics, Beijing Institute of Technology, Beijing 100081, China; jiangwei.shang@bit.edu.cn (J.S.); berge@bit.edu.cn (B.-G.E.); 4 Department of Physics, National University of Singapore, Singapore 117542, Singapore; 5 Centre for Quantum Technologies, Singapore 117543, Singapore; cqtnhk@nus.edu.sg (H.K.N.)

**Keywords:** quantum state sampling, sequential Monte Carlo, sequentially constrained Monte Carlo, Markov chain, bound entanglement, positive partial transpose, computational cross norm, realignment, curse of dimensionality, target distribution, Wishart distribution, 02.50.Ng, 03.65.Aa, 03.65.Ud, 03.65.Wj

## Abstract

Random samples of quantum states with specific properties are useful for various applications, such as Monte Carlo integration over the state space. In the high-dimensional situations that one already encounters when working with a few qubits, the quantum state space has a very complicated boundary, and it is challenging to incorporate the specific properties into the sampling algorithm. In this paper, we present the Sequentially Constrained Monte Carlo (SCMC) algorithm as a practical and versatile method for sampling quantum states in accordance with properties that can be stated as inequalities. We apply the SCMC algorithm to the generation of samples of bound entangled states; for example, we obtain nearly ten thousand bound, entangled, two-qutrit states in a few minutes, compared with less than ten such states per day from independence sampling in our implementation. In the second application, we draw samples of high-dimensional quantum states from a narrowly peaked target distribution and observe, for the system sizes investigated, that SCMC sampling remains computationally manageable as the dimensions grow. In yet another application, the SCMC algorithm produces uniformly distributed quantum states in regions bounded by values of the problem-specific target distribution; such samples are needed when estimating parameters from the probabilistic data acquired in quantum experiments.

## 1. Introduction

Random samples of quantum states with specific probability distributions and/or governed by specific physical properties are very useful for many applications in quantum information science and other research areas. For instance, they play an important role in quantum state and parameter estimation [1,2,3], allowing one to test and verify properties for certain classes of quantum states [4,5,6] as well as to test quantum channels and processes, such as the random quantum circuits leading to Quantum Supremacy [7,8,9]. Similar to sampling classical systems, independent rejection sampling methods are often inapplicable to high-dimensional quantum systems as they suffer severely from the exponential growth of resource requirements, known as the “curse of dimensionality” [10]. The positivity boundaries of the quantum state spaces, moreover, are typically extremely complex to characterize for otherwise convenient parameterizations, causing quantum states to be more challenging to sample.

Most existing methods for the direct sampling of the random density matrices, which represent the quantum states numerically, rely on either clever parameterization of the state space or various kinds of induced measures [11,12,13,14,15]. The major drawback is that the exact probability distributions for random states constructed this way are usually not known; one has to estimate approximate distributions, and achieving this reliably is a numerical challenge in itself. Thus, rejection sampling methods that proceed from known proposal distributions are often still favored for sampling from a desired target distribution. However, even if one can directly draw from a reliable reference distribution that mimics the target distribution well—for example, using a version of the complex Wishart distribution [16,17,18]—the exponential reduction of the acceptance rate as the dimensions increase makes it rather impractical to go beyond sampling small quantum systems (see, for example, Reference [19]); other rejection sampling-based methods, such as adaptive rejection sampling, suffer from this problem, too.

Monte Carlo-based algorithms [20] can be much more resilient to this curse of dimensionality. The major difficulty encountered in using Monte Carlo (MC) methods comes from the incorporation of constraints into the parameter space. For instance, enforcing the positivity constraint needed for a physical state, which is intrinsic to quantum systems, can be extremely challenging. Algorithms that rely on clever random walks in the probability simplex, such as the Markov Chain Monte Carlo (MCMC), suffer from low acceptance rates because of this positivity constraint, and the Hamiltonian Monte Carlo (HMC) algorithm [21], whose random walk stays in the state space, depends on the evaluation of a Jacobian determinant and its derivatives, found to be numerically unreliable in high-dimensional quantum state spaces [22,23].

In this work, we investigate the use of Sequentially Constrained Monte Carlo (SCMC) samplers as a practical tool for sampling quantum states in accordance with a given distribution and/or certain physical properties, with particular emphasis on efficiently enforcing quantum constraints. SCMC samplers were first proposed by Golchi and Campbell in 2016 [24] to effectively impose constraints when sampling classical systems as an extension to the Sequential Monte Carlo (SMC) samplers proposed by Del Moral et al. a decade earlier [25]. SMC methods [26,27], which have been used extensively in the context of sequential Bayesian inference, are not new to the field of quantum information. For example, they have been well used in adaptive/online Bayesian Hamiltonian estimation [3,28,29]. In the context of sequential Bayesian inference, the sequence of distributions is constructed as more and more measurement data are taken into consideration. Unlike SMC methods in Bayesian inference, SMC samplers enable efficient sampling using MCMC algorithms through the introduction of a sequence of artificial intermediate distributions that bridge between an easy-to-sample initial distribution and the difficult-to-sample target distribution. One application of SMC samplers in quantum information science was recently presented in [30]. Here, we employ SMC samplers to sample quantum states, making use of SCMC samplers to better incorporate the constraints that naturally arise in quantum problems.

We study the performance of SCMC samplers for sampling quantum states through three concrete examples, which are otherwise difficult, or even impractical, to achieve using the standard existing methods mentioned above. In the first example, we show how SCMC samplers can be applied to sample quantum states with bound entanglement through the imposition of soft constraints. Large samples of uncorrelated bound entangled bipartite systems with dimensions 3×3, 3×4, 4×4, and 3×5 are reported, and a curious property of the 2×4 system is observed. In this context, we note that the SCMC algorithm does not rely on a particular parameterization of the bound entangled state; see Section 3.1.

The second example presents a generic approach to sampling quantum states from desired target distributions. The efficiency and reliability of our algorithm is demonstrated via sampling three-qubit and four-qubit states. Moreover, within the range of system sizes considered, the results are consistent with a computational cost that grows much more mildly than what is typically encountered in direct rejection sampling.

In the last example, we sample uniformly distributed quantum states in regions bounded by the contours of the target distribution—uniform with respect to the volume elements induced by the Hilbert–Schmidt distance, that is. We improve on the implementation of the method recently introduced by Oh, Teo, and Jeong (OTJ) [31,32] and so confirm that the direct SCMC sampling from the target distribution is reliable. In addition, we observe that direct SCMC sampling is more efficient than the indirect OTJ method. Depending on one’s point of view, the last example can be regarded as benchmarking SCMC against OTJ or OTJ against SCMC.

Some technical details are reported in the Appendix. Selected samples and codes can be fetched from a dedicated repository [33].

## 2. SCMC Sampling

The SMC samplers presented in Reference [25] enable one to sample x efficiently from its difficult-to-sample target distribution f(x) through a sequence of intermediate distributions. We choose an initial distribution g(x), which can be the prior or any appropriate reference distribution that is easy to sample from directly, and find a discrete sequence of $N_{\tau }$ density distributions ${\left(h_{i}\left(x\right)\right)}_{i=0}^{N_{\tau }}$ that smoothly bridges between g(x) and f(x). One particular choice of $h_{i}\left(x\right)$ is to follow the geometric path [34,35],

(1) $$h_{i}\left(x\right)=f{\left(x\right)}^{\tau_{i}}g{\left(x\right)}^{\left(1-\tau_{i}\right)}\;,$$

where $\tau_{i}$ runs from 0 to 1 in arithmetic progression, i.e., $\tau_{i}=i/N_{\tau }$ as *i* goes from 0 to $N_{\tau }$. Thus, $h_{0}\left(x\right)=g\left(x\right)$ and $h_{N_{\tau }}\left(x\right)=f\left(x\right)$.

The utility of SMC samplers was extended by Golchi and Campbell [24] to SCMC samplers; in their approach the hard constraints on the parameter space are gradually incorporated into the intermediate distributions as soft probabilistic constraints. Here, soft constraints refer to relaxations that approach the hard constraint in the limit. For instance, when the desired hard constraint is κ(x)>0, with its indicator function being the step function $I_{\kappa }\left(x\right)=\eta \left(\kappa (x)\right)$, the intermediate indicator functions for the soft constraints can be smooth approximations of the Heaviside step function, such as

(2) $$I_{\kappa ,i}\left(x\right)=\frac{1+\tanh\left(a\tau_{i}\kappa \left(x\right)\right)}{2}\;,$$

where the *tolerance a* is an adjustable scale controlling the *hardness* $a\tau_{i}$ of the constraint. In this case, the indicator function $I_{\kappa ,i}\left(x\right)$ is incorporated into the SCMC sampler by taking

(3) $$h_{i}\left(x\right)\to h_{i}\left(x\right)I_{\kappa ,i}\left(x\right)\;,$$

and, within the limit of $a\tau_{i}\to \infty$, it converges to the hard constraint, i.e.,

(4) $$I_{\kappa ,N_{\tau }}\left(x\right)\to \left\{\begin{array}{cc} 1 & \kappa (x)>0\;,\\ 0 & \kappa (x)<0\;. \end{array}\right.$$

In practice, for a large but yet finite value of *a*, points with κ(x)≤0 (κ(x)>0) are rejected (accepted) with a higher and higher probability as $\tau_{i}\to 1$; thus, sample points gradually move towards the region satisfying the constraint. At the final step of the SCMC algorithm, we impose the hard constraint instead of the probabilistic constraint and reject the sample points that violate the constraint. Between consecutive distribution steps, the sample is propagated by $N_{\mathrm{MC}}$ MCMC iterations, while the effective sample size is monitored to determine whether resampling is needed. We set $N_{\mathrm{MC}}$ sufficiently large, relative to the observed MCMC acceptance rate, so that each sample point is likely to move at least once between resampling events; once this condition is met, further increasing $N_{\mathrm{MC}}$ mainly increases CPU time and reduces residual correlations without noticeably changing the final sample in our examples. Consult the Appendix for implementation details.

The ability to enforce constraints via SCMC is very useful for sampling quantum states because the quantum Hilbert space intrinsically requires the physicality constraint, which usually means complicated constraints on the parameters (positivity of a large matrix) that are CPU expensive to check. Apart from the physicality constraint, many other interesting quantum constraints can also be extremely difficult to enforce as suitable parameterizations of the states are unavailable. For efficient sampling of quantum states, we need to identify the quantum constraints and find their corresponding indicator functions. Next, we show how well the SCMC sampler works for sampling quantum states in three distinct contexts.

## 3. Examples

### 3.1. Bound Entanglement

A bound entangled state is a state with non-distillable entanglement. It has drawn a lot of attention in the quantum information community since the prediction of its existence in 1998 [36]; see [37] for a recent review. Random samples of bound entangled states are useful not only for the field of quantum information but also for mathematical interests. On the one hand, states with bound entanglement provide a testbed for studying the relationship between entanglement, steering, and Bell-type nonlocality [38,39]; on the other hand, bound entangled states enable the study of positive maps from a different perspective [40]. Moreover, despite being highly mixed, such states are potentially useful for quantum cryptography and quantum metrology [41,42].

While it is currently unknown if all bound entangled states have a positive partial transpose (PPT), we do know that all entangled PPT states are bound entangled [36], and a PPT state is surely entangled if the computable cross norm or realignment (CCNR) criterion is met [43]. Taken together, then, the two conditions

(5) $$\min\left\{\mu_{\mathrm{PT}}\right\}\geq 0\;\mathrm{and}\;\sum_{j}\sigma_{j}\left(\tilde{\rho }\right)\equiv R>1$$

are sufficient to ensure bound entanglement. Here, $\left\{\mu_{\mathrm{PT}}\right\}$ is the set of eigenvalues of the partial transpose of the density matrix ρ, and *R* is the sum of the singular values of the realigned matrix $\tilde{\rho }$; see Equation (5) in Reference [43]. Examples of bound entangled states are studied in the literature; see, for example, References [36,38,44,45,46,47,48,49,50]. They are often given as special constructions of states in some particular parameter families. A more general way of constructing bound entangled states which allows the generation of a random sample has also been presented in Reference [51], but it only works for bipartite systems with equal dimensions.

**Figure 1:**
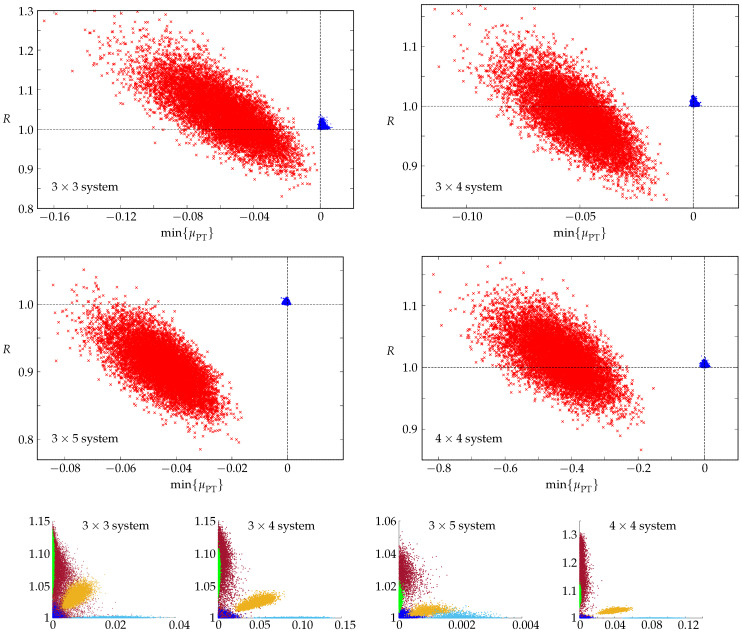
The generation of bound entangled states using SCMC. The initial reference sample has $10^{4}$ states (red crosses) uniformly distributed with respect to the Hilbert–Schmidt distance shown here on an *R* vs. $\min\{\mu_{\mathrm{PT}}\}$ plot. The states after SCMC are indicated by the blue dots. Out of these 10,000 post-SCMC states, 8530, 7011, 2211, and 4013 states are bound entangled for the respective systems of dimensions 3×3, 3×4, 3×5, and 4×4. The plots in the bottom row show samples in the first quadrant, where the double criterion of Equation (5) is obeyed, filtered through further MCMC iterations, with different colors representing different propagation kernels (by choosing different directions of the random walks).

The SCMC samplers are well suited for generating random samples of PPT bound entangled states with no restrictions on the states’ parameter family or on their dimensionality. They can be generated by imposing the two criteria in Equation (5) in the same way as one imposes the physicality constraint. The two indicator functions are in the form of Equation (2) with $\kappa_{1}\left(\rho \right)=\min\left\{\mu_{\mathrm{PT}}\right\}$ and $\kappa_{2}\left(\rho \right)=R-1$, respectively. The initial reference sample of dimension *d* can be drawn directly from a uniform distribution of physical states using the Wishart distribution $W_{d}^{\left(Q\right)}\left(d,1_{d}\right)$, that is, they are generated from d×d normally distributed complex matrices with mean zero and covariance matrix $1_{d}⊗1_{d}$ (see Reference [19] for more details). Since the reference samples are physical initially, and we do not want to have unphysical sample points as the samples are processed, we impose hard physicality constraints during the Markov chain steps to ensure that the random walks do not move a quantum state beyond the physical boundary. Examples illustrating the generation of random samples of bound entangled states are shown in Figure 1, where the states in the initial reference sample are represented by red crosses and the sample states after SCMC and before the final rejection sampling are represented by blue dots. As is clearly visible from the plots, our SCMC sampler successfully moved the states toward the region around R=1 and $\min\{\mu_{\mathrm{PT}}\}=0$ and produced bound entangled states. Table 1 lists the parameters used for the SCMC sampling together with the yield of bound entangled states and the CPU time consumed in each distribution step.

As discussed in the previous section, the efficiency of SCMC depends on a number of parameters, including the tolerance $a_{p}$ for the PPT constraint, the tolerance $a_{e}$ for the CCNR entanglement constraint, and the number $N_{\tau }$ of the distribution steps. These tuning parameters are strongly problem dependent; their useful ranges vary with the dimension, target distribution and the particular constraints, as is also reflected by the wide ranges for $a_{e}$ and $a_{p}$ in Table 1. In this feasibility study, we therefore select them empirically, using the final rejection yield and the computational time as practical diagnostics rather than attempting a universal optimization rule.

Rejection sampling is applied to impose the complete set of hard constraints at the end of the algorithm; thus, a good set of the parameters should result in a high yield of accepted sample points within reasonable CPU time. For the bipartite qutrit system of dimension d=3×3, a 99% yield of bound entangled states was obtained by setting $N_{\tau }=300$ and $a_{e}=a_{p}=5\times 10^{4}$. On our standard desktop, it took a few minutes to find thousands of two-qutrit bound entangled states; CPU parallelization is possible and will speed up the computation. For comparison, we conducted independent sampling by drawing $10^{10}$ states from a uniform distribution of bipartite qutrit states. Only 24 out of the $10^{10}$ states obeyed the criteria of Equation (5), and this process took days even with CPU parallelization. Such a search for higher-dimensional systems (3×4, 3×5, or 4×4, say) is even more difficult. This illustrates a substantial practical improvement provided by the SCMC algorithm for this task.

**Figure 2:**
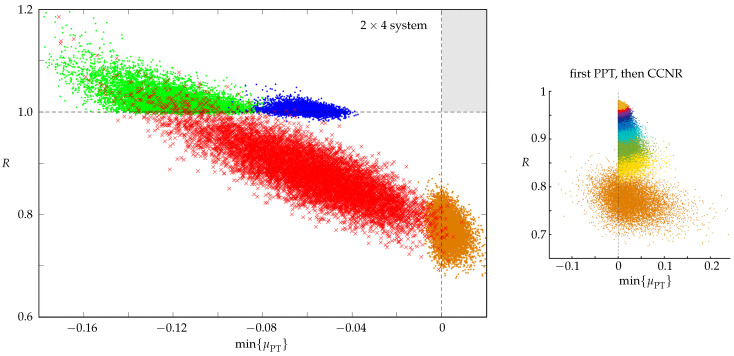
Search for bound entangled states with SCMC for the 2×4 system. **Left:** The initial reference sample has $10^{4}$ states (red crosses) uniformly distributed with respect to the Hilbert–Schmidt distance shown here on an *R* vs. $\min\{\mu_{\mathrm{PT}}\}$ plot. The states after SCMC are indicated by the blue dots. There are no blue dots in the first quadrant (gray) where the criteria (5) are obeyed. Enforcing only the PPT criterion results in the orange sample; enforcing only the CCNR criterion results in the green sample. **Right:** The orange sample is shown together with, in different colors, further samples that are obtained by SCMC steps toward enforcing the CCNR criterion.

In Figure 1 and Figure 2, we show scatter plots of the $\min\{\mu_{\mathrm{PT}}\}$ and *R* values of samples obtained by SCMC for five systems with Hilbert-space dimensions between eight and sixteen (state space dimensions between 63 and 255). No exhaustive trials were conducted, and the performance can certainly be improved upon, as we did not optimize the parameters. Since we are imposing the constraints through the two inequalities in Equation (5), the states produced were clustered in the small corner near R=1 and $\min\{\mu_{\mathrm{PT}}\}=0$ for the four systems in Figure 1, but not for the 2×4 system in Figure 2. For the cases of Figure 1, to explore other parts of the space for larger values of *R* and $\min\{\mu_{\mathrm{PT}}\}$, we filtered the sample further through MCMC iterations with different kernels and acceptance criteria, such as “accept only if *R* is increased;” the samples obtained are shown in the the bottom row of Figure 1. This way of producing random samples of bound entangled states, without relying on special constructions of parameter families of states, will be very useful when studying general properties of bound entanglement; such investigations are, however, not the objective of this work.

The 2×4 system of Figure 2 is particular—we could not find any states that satisfy both the PPT and the CCNR criterion in (5). When either one of the criteria is enforced, SCMC yields samples with $\min\{\mu_{\mathrm{PT}}\}\geq 0$ or R>1, respectively. As the plot on the right in Figure 2 shows, first enforcing the PPT criterion, followed by SCMC steps toward enforcing the CCNR criterion, produces samples with *R* values that are sequentially closer to the R=1 threshold without, however, crossing it. We conjecture that 2×4 systems do not have bound entangled states that obey the sufficient double criterion of (5). There are, of course, bound entangled states that do not obey these criteria. Indeed, the families of bound entangled 2×4 states constructed by the authors of [44,47,48] are PPT with R≤1; their values are on the straight lines with endpoints $(\min\left\{\mu_{\mathrm{PT}}\right\},R)=\left(0,1\right)$ and (0,0.7866), (0,0.8727) and (0.0270,0.7572), or (0,1) and (0,0.8536), respectively. In Figure 2, they are all on, or very close to, the dashed vertical line at $\min\{\mu_{\mathrm{PT}}\}=0$.

These examples illustrate that the criteria (5) are not necessary—the pair is sufficient. It is possible, perhaps likely, that the final SCMC samples (blue dots in Figure 1 and Figure 2) contain bound entangled states outside the first quadrant, but the yields reported in Table 1 refer solely to the states that meet the double criterion.

### 3.2. Desired Target Distribution

Random samples from a desired distribution are useful in various contexts in quantum information science such as studying properties of a quantum system, parameter optimization, or model testing. Owing to the notorious curse of dimensionality and/or quantum constraints, sampling methods that work well for low-dimensional systems fail to work in practice as the dimensions increase. (The curse refers to the dimensionality of the parameter space, not of the Hilbert space.) For example, the generation of a target sample from a uniform reference distribution of states through rejection sampling fails to work for three-qubit systems, as the acceptance rate is extremely low; one can increase the acceptance rate by orders of magnitude by replacing the uniform reference sample with an appropriate Wishart distribution of states. Nevertheless, the efficiency still suffers from an exponential decay with respect to dimensionality [19]. On the other hand, Markov chain algorithms suffer from instability and their unavoidable sample correlation [22,23]. In this section, we demonstrate that SCMC can be used for generating samples of a desired target distribution at high dimensions, impractical otherwise, and, for the system sizes investigated, the computational cost does not show the severe exponential deterioration characteristic of direct rejection sampling.

To be specific, we consider the generic situation where the desired distribution is of the Dirichlet form

(6) $$f\left(\rho \right)\propto \prod_{k}\mathrm{tr}{\left(\Pi_{k}\rho \right)}^{\alpha_{k}}=\prod_{k}p_{k}^{\alpha_{k}},\;\rho >0\;,$$

which is central to quantum state estimation with a conjugate prior. The probability operators $\Pi_{k}$ have the usual properties, namely $\Pi_{k}\geq 0$ and $\sum_{k}\Pi_{k}=1$, and the set $\Pi =\{\Pi_{k}\}$ is an informationally complete measurement so that there is a one-to-one correspondence between the state ρ and the probabilities $p=\left\{p_{k}\right\}=\mathrm{tr}\left(\Pi \rho \right)$. Note that a sample drawn from the Dirichlet distribution by one of the standard efficient algorithms, which draw from the probability simplex, has many unphysical entries ($p_{k}>0$ while ρ≱0), and simply discarding them would results in a very poor yield. The SCMC procedure pushes most of the initially unphysical sample points into the physical state space. Similar remarks apply to samples drawn from a Dirichlet distribution centered at the state for which f(ρ) is largest, which is often a rank-deficient state. Note also that the Dirichlet distribution has a single very narrow peak when $A=\sum_{k}\alpha_{k}$ is large, as is the typical situation in quantum state estimation scenarios.

To apply SCMC, we first need a set of initial sample points that can be easily generated. Conventional MC methods often use samples generated in the probability simplex, such as the Dirichlet distribution, that do not respect the positivity constraints, but the rate of physical samples decreases exponentially with respect to the dimensions of the parameter space, which makes it impractical for high-dimensional systems (for example, the physical rate is less than $10^{-10}$ for a three-qubit system [19]). Therefore, for efficient sampling, it is expedient to use an initial reference sample that is physical. We mainly explore two types of initial reference sample distributions: the uniform distribution with respect to the Hilbert–Schmidt distance and the peaked Wishart distribution. The former rarely resembles any feature of the target distribution, but the intermediate distributions $\left\{h_{i}\left(\rho \right)\right\}$ are straightforward to evaluate for each sample point in the algorithm. The Wishart distribution for quantum states $W_{d}^{\left(Q\right)}\left(n,\Sigma \right)$ offers more freedom by adjusting the covariance matrix Σ in shaping the reference distribution towards a target while conforming to the physicality constraint. Its probability density for a *d*-dimensional system is [19]

(7) $$g\left(\rho \right)\propto \frac{\det{\left(\rho \right)}^{n-d}}{\mathrm{tr}{\left(\Sigma^{-1}\rho \right)}^{nd}}\;,$$

where $\rho =Z^{†}Z/\mathrm{tr}\left(Z^{†}Z\right)$ and the d×n random complex matrix *Z*, with n≥d, is drawn from a Gaussian distribution with zero mean and covariance matrix $1_{n}⊗\Sigma$; we get the uniform distribution for n=d and $\Sigma =1_{d}$. We expect faster convergence using the Wishart distribution, as it is more similar to the target distribution. However, the apparent downside is that the numerical evaluation of its initial and resulting intermediate distributions takes longer.

To test its performance, we run the SCMC algorithm for different numbers of intermediate distributions $N_{\tau }$ and different types of initial reference distributions. We assess the quality of the sample by a variant of the method described in Section 5 of Reference [19], for which we introduce the nested λ-regions with f(ρ)≥λF, where $F=\max_{\rho }\left\{f(\rho )\right\}$ is the peak value of f(ρ), and 0≤λ≤1. We have the full physical state space for λ=0 and only the peak location for λ=1. The fraction of the target distribution contained in a λ-region is its *content* $c_{\lambda }$:

(8) $$c_{\lambda }=\int \left(d\rho \right)\;f\left(\rho \right)\eta \left(f(\rho )-\lambda F\right)\;,$$

where $c_{\lambda =0}=1$ reflects the normalization of f(ρ) to unit integral. By counting how many sample points are inside a λ-region, we obtain an estimate for the respective $c_{\lambda }$ value. Note that, in the context of Bayesian estimation, $c_{\lambda }$ is the credibility of the region, if f(ρ) is the posterior distribution.

**Figure 3:**
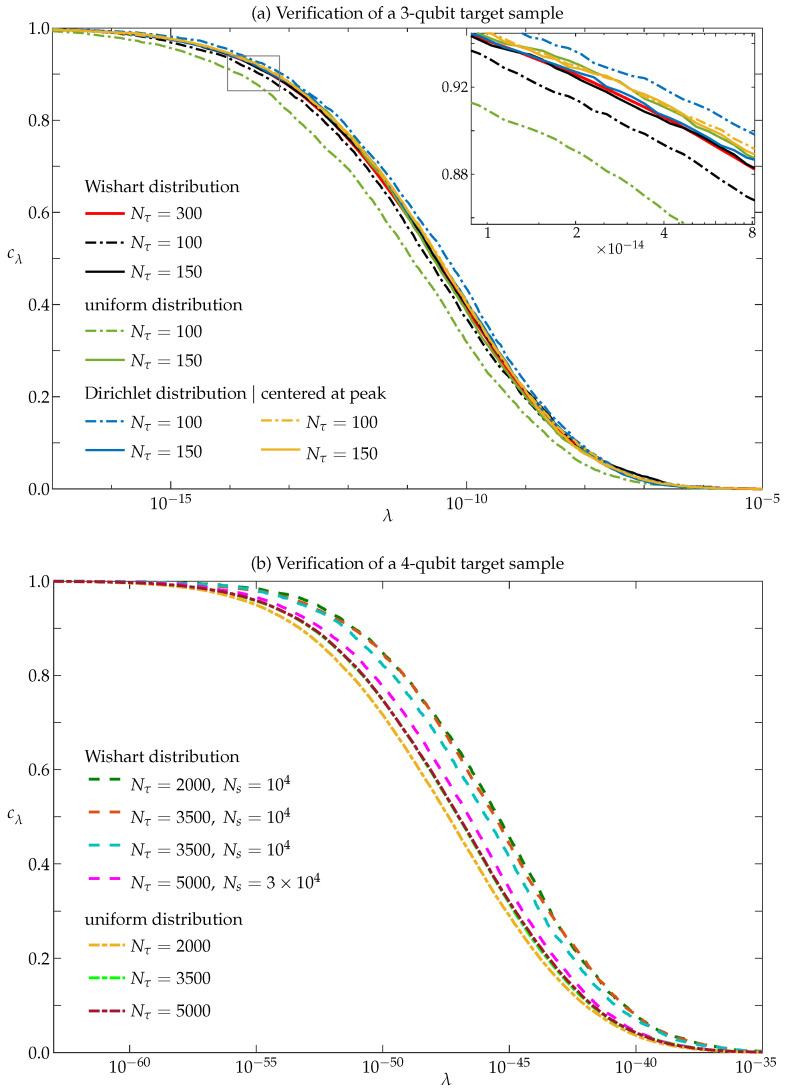
The content $c_{\lambda }$ evaluated for samples generated for (**a**) a three-qubit target distribution and (**b**) a four-qubit target distribution. The target distributions are given by A=3000 randomly generated detection events for product tetrahedron measurements. The samples are obtained using SCMC with $N_{s}$ initial reference points drawn from the Wishart distribution, the uniform distribution, the Dirichlet distribution, or the Dirichlet distribution centered at the peak of f(ρ). The SCMC algorithm is run for different numbers of intermediate distributions $N_{\tau }$. The inset in (**a**) is a blow-up of the marked rectangular area. In lexicographic order, the counts of the simulated product tetrahedron measurements detection events that make up the data for the target distributions are as follows: for the three-qubit example, {36, 13, 64, 71, 14, 16, 7, 15, 60, 10, 84, 63, 64, 9, 55, 71, 8, 12, 10, 16, 16, 48, 67, 62, 9, 64, 75, 63, 10, 74, 60, 73, 65, 14, 62, 66, 9, 57, 76, 53, 82, 78, 128, 22, 61, 44, 25, 27, 56, 12, 52, 66, 14, 76, 56, 78, 45, 47, 22, 27, 66, 68, 25, 102}; and the four-qubit example, {5, 5, 11, 9, 5, 6, 3, 5, 11, 4, 18, 18, 11, 3, 10, 24, 5, 4, 2, 5, 1, 5, 1, 2, 2, 1, 3, 1, 5, 6, 3, 4, 16, 3, 24, 15, 3, 4, 4, 0, 16, 4, 27, 14, 13, 1, 17, 20, 9, 4, 16, 16, 4, 2, 2, 4, 21, 3, 27, 23, 19, 4, 18, 35, 2, 3, 1, 6, 4, 0, 4, 5, 4, 6, 7, 4, 3, 3, 1, 6, 0, 4, 4, 4, 0, 15, 14, 5, 2, 5, 19, 17, 3, 8, 14, 12, 2, 2, 2, 2, 2, 16, 13, 13, 3, 26, 26, 21, 7, 27, 13, 13, 2, 1, 2, 4, 2, 8, 17, 21, 2, 23, 21, 13, 4, 24, 21, 18, 7, 5, 17, 19, 4, 1, 9, 1, 10, 3, 25, 17, 19, 4, 15, 18, 1, 6, 1, 1, 3, 15, 15, 17, 3, 20, 26, 21, 1, 15, 21, 17, 14, 5, 41, 16, 10, 14, 40, 23, 35, 22, 71, 20, 14, 17, 22, 3, 13, 0, 16, 17, 1, 9, 12, 23, 16, 17, 20, 1, 14, 27, 2, 17, 12, 3, 17, 20, 4, 6, 4, 3, 17, 4, 19, 13, 17, 3, 14, 21, 5, 5, 4, 6, 3, 13, 11, 18, 1, 17, 20, 19, 2, 20, 19, 26, 21, 4, 20, 21, 3, 24, 22, 14, 18, 17, 22, 4, 15, 17, 6, 14, 18, 6, 11, 21, 4, 24, 18, 28, 18, 16, 0, 21, 30, 28, 11, 67}.

We expect that this estimate is better (i) when more intermediate steps $N_{\tau }$ are used in the SCMC algorithm and (ii) when the sample size $N_{s}$ is larger. Both expectations are confirmed by the data presented in Figure 3 for two examples, one for a three-qubit system and one for a four-qubit system. The two f(ρ)s are posterior distributions (for a uniform prior) for A=3000 randomly generated measurement clicks of product tetrahedron measurements.

With this large *A* value, the distributions are squeezed into a tiny region in the immediate vicinity of the peak location of f(ρ) at or near the boundary of the state space, which makes them impractical for sampling with conventional methods. In the SCMC algorithm, besides imposing the sequence of intermediate distributions to approach the desired one gradually from the reference distribution, the physicality constraints also have to be imposed either strictly or gradually. When the initial sample points are guaranteed to be physical, such as the ones uniformly drawn from the state space, the hard physicality constraint is directly imposed during the MCMC iterations. Otherwise, when a portion of the initial sample distribution is unphysical, as in the case of the linearly shifted Wishart distribution [19] or the Dirichlet distributions, we impose the soft physicality constraint gradually along with the distribution steps. Note that, when the constraint is strictly imposed throughout, one could make use of a Cholesky decomposition to check for a semi-positive-definite and hermitian matrix; then, the positivity of the smallest eigenvalue of the matrix corresponding to ρ is used to impose the soft constraint via the indicator function.

In the three-qubit example shown in Figure 3a, we find it sufficient to have $N_{\tau }=300$ intermediate steps when the initial sample is drawn from an appropriate Wishart distribution, as the content barely changes by increasing $N_{\tau }$ further. When $N_{\tau }=100$, the Dirichlet distribution centered at the peak of f(ρ) performs the best, and the uniform distribution performs the poorest. Their difference becomes less and less noticeable with increasing number of distribution steps. For example, no significant difference is shown in their performance when $N_{\tau }=150$. Thus, it is evident that the SCMC algorithm tolerates flexibility in the choice of the initial reference distribution, although a reference distribution that better resembles the target can offer faster convergence.

Figure 3b shows the content of the four-qubit samples generated starting from either a Wishart distribution or a uniform distribution. The Dirichlet distribution is impractical to use here because the random walk into the physical space—a tiny subspace within the 255-dimensional probability simplex—is extremely inefficient. For the same number of sample points, the Wishart distribution might appear to provide faster convergence than the uniform distribution in terms of $N_{\tau }$. However, the overall performance using the uniform distribution is practically better. This is because the computation per sample point is about three to four times faster when using the uniform distribution than for the Wishart distribution. A larger sample size helps not only to reduce the statistical error in evaluating sample average quantities like the content; it also makes the random walk, which is set to propagate along the direction given by the covariance matrix of the current sample, more efficient, as the covariance matrix is more accurately estimated when the sample is larger. As a result, the time cost for using the Wishart distribution with $\left\{N_{s}=3\times 10^{4},N_{\tau }=3500\right\}$ is about the same as using the uniform distribution with $\left\{N_{s}=10^{5},N_{\tau }=3500\right\}$, but the latter converges better, as is visible in Figure 3b.

The computational time roughly scales as $O(N_{\tau }\sqrt{N_{s}})$ with simple vectorization of the code in Python (version 3.5.7), provided that there is sufficient memory. For the examples shown in Figure 3, the sampling of $10^{5}$ three-qubit states and $N_{\tau }=200$ distribution steps took about $2\times 10^{4}$ s using the Wishart distribution on a regular desktop with no CPU parallelization and $7\times 10^{3}$ s using the uniform distribution. The sampling of $10^{5}$ four-qubit states and $N_{\tau }=3500$ distribution steps using the uniform distribution, which showed good convergence, took about $2.2\times 10^{5}$ s (∼60 h, which is only about 30 times longer than sampling three-qubit states). We also ran the sampling algorithm for one-qubit and two-qubit states with reliable sample verification. Using the uniform distribution, the sampling of $10^{5}$ one-qubit states and $N_{\tau }=10$ takes about 260 s, and the sampling of $10^{5}$ two-qubit states and $N_{\tau }=75$ takes about $1.6\times 10^{3}$ s. Similar scaling of running time was seen in other distributions we sampled. All sampling is conducted on a standard desktop with 8 GB RAM.

In summary, for the SCMC sampling from the state spaces for one to four qubits, which have (3,15,63,255) parameters, the number of required distribution steps is (10,75,200,3500)$≃\left(3^{2.1},15^{1.6},63^{1.3},255^{1.5}\right)$, and the computational time is roughly $\left(3^{1.3},15^{1.2},63^{1.2},255^{1.5}\right)$ minutes. The number of distribution steps required for convergence increases as the dimensionality increases, leading to longer computational time, and additional CPU cost is incurred by the multiplication of ever larger matrices. The number of distribution steps, $N_{\tau }$, is a problem-dependent tuning parameter: Increasing $N_{\tau }$ makes the continuation between intermediate distributions more gradual and gives greater confidence in convergence, but it also increases the running time approximately linearly. In practice, we choose $N_{\tau }$ through short trial runs that balance the observed stabilization of the sample, such as the stabilization of the estimated content, against available computational resources.

For the scaling of the running time, we observe exponents that are approximately independent of the dimension examined, and this suggests that—*within this tested range*—the SCMC algorithm does not exhibit the exponential increase in computation time with respect to dimension that hampers direct rejection sampling. Put differently, the SCMC sampler remains computationally practical as the dimensionality increases, at least for dimensions up to 255. Owing to limited time and computer memory, we did not sample larger quantum systems. However, a powerful work station running the CPU-parallelization version of the code should be able to not only produce a larger sample in a much shorter period of time but also sample quantum states in higher dimensions. These results indicate that further exploration of SCMC in higher-dimensional settings would be worthwhile.

### 3.3. The Oh–Teo–Jeong Method for Benchmarking

The volume element (dρ) in Equation (8) is also the probability element of the uniform distribution, and 

(9) $$s_{\lambda }=\int \left(d\rho \right)\;\eta \left(f(\rho )-\lambda F\right)$$

is the *size* of the λ-region, normalized such that $s_{\lambda =0}=1$. The relation [52]

(10) $$c_{\lambda }=\frac{\lambda s_{\lambda }+\int_{\lambda }^{1}d\lambda^{\prime }\;s_{\lambda^{\prime }}}{\int_{0}^{1}d\lambda^{\prime }\;s_{\lambda^{\prime }}}$$

can be used to obtain an estimate of $c_{\lambda }$ from an estimate of $s_{\lambda }$. Owing to the very narrow peak of f(ρ), however, we cannot estimate $s_{\lambda }$ by counting how many points of a uniform sample are in the λ-region—there would be far too few sample points in the vicinity of the peak.

The OTJ method introduced in [31,32] offers a clever solution to this problem based on their region-average computation lemma. It provides $s_{\lambda }$ by an integration,

(11) $$s_{\lambda }=s_{\lambda_{0}}\frac{g_{\lambda_{0}}}{g_{\lambda }}\exp\left(-\int_{\lambda_{0}}^{\lambda }\frac{d\lambda^{\prime }}{\lambda^{\prime }g_{\lambda^{\prime }}}\right)\;,$$

where $g_{\lambda }$ is the average value of $\ln\left(f(\rho )/(\lambda F)\right)$ over the λ-region with respect to the uniform distribution. Although f(ρ) is very narrowly peaked, $\ln\left(f(\rho )\right)$ is not, and $g_{\lambda }$ can be estimated accurately by a sufficiently large sample drawn from the uniform distribution over the λ-region, not over the whole quantum state space. Accordingly, an implementation of the OTJ method requires an accurately known reference value $s_{\lambda_{0}}$ and a reliable procedure for generating a uniform sample for each λ-region. Both ingredients had problems in the implementation reported in [31,32].

Here, we implement the OTJ method by (i) finding $s_{\lambda_{0}}$ from a large uniform sample for $\lambda_{0}$ small enough that, at minimum, a few percent of the sample points are in the $\lambda_{0}$-region and (ii) generating large uniform samples from successive λ-regions using an SCMC algorithm that gradually imposes the constraint f(ρ)≥λF. For the computation of the $\lambda^{\prime }$ integral in Equation (11), we discretize linearly in $\ln(\lambda^{\prime })$.

**Figure 4:**
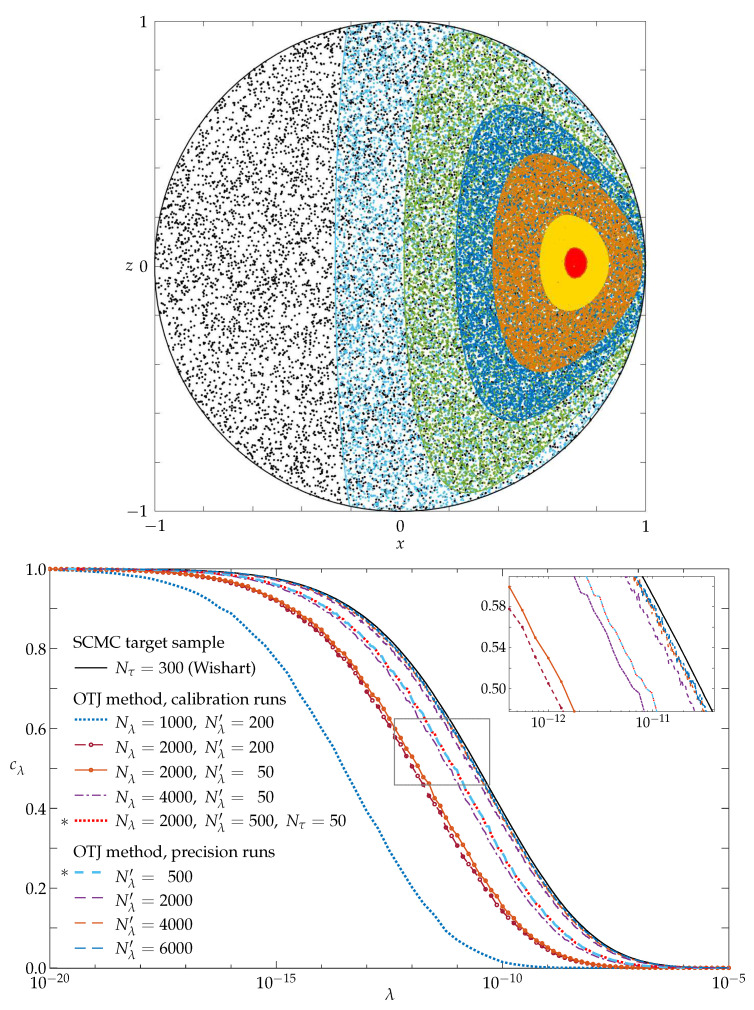
OTJ algorithm illustrated. **Top:** Uniform samples on the λ-regions for single-qubit states on the equatorial disk of the Bloch ball. The target distribution refers to the experimental data of a distorted trine measurement [54] with $\left(\alpha_{1},\alpha_{2},\alpha_{3}\right)=\left(1802,315,303\right)$ in Equation (6). The scattered points of different colors mark $10^{4}$ states each in the λ-regions with $\log_{10}\left(\lambda \right)=-\infty$, −400, −200, −100, −50, −10, and −1, respectively. The initial distribution (λ=0, black points) is uniform on the disk. **Bottom:** Content $c_{\lambda }$ evaluated for the three-qubit target distribution of Figure 3a. The black curve is computed directly from a target sample with $N_{s}=10^{6}$ points generated from a Wishart sample by SCMC. The other colored curves show results from several runs of the OTJ algorithm. The inset is a blow-up of the marked rectangular area.

Figure 4 illustrates aspects of the OTJ algorithm. At the top we see that the λ-regions are not of ellipsoidal shape—the “accelerated hit-and-run” algorithm used in [31,32] assumes that the λ-regions have ellipsoidal shape, which is only justified for λ≲1, no such assumptions enter the SCMC algorithm—and that one needs truly tiny positive λ values to enclose a sizable fraction of a uniform sample on the whole state space. The situation is that of a three-outcome measurement in which A=2420 events were observed; see p. 224 in Reference [53]. While this is not a lot of data for a one-qubit measurement, the Bayesian posterior (our target function here) is already peaked extremely narrowly.

At the bottom in Figure 4, we show $c_{\lambda }$ for a three-qubit target distribution obtained from sampling directly from the distribution by SCMC (black curve) and by different runs of the OTJ algorithm (colored curves). Figure 4 (bottom) confirms that the SCMC sampling yields the correct $c_{\lambda }$ values, as the sequence of OTJ curves converges toward the SCMC curve. This convergence is from below, which tells us that the OTJ estimates of $c_{\lambda }$ have a negative bias.

All SCMC runs of the OTJ method proceed from λ=1 when all sample points are at the peak location. By reducing λ in $N_{\lambda }$ steps, we obtain uniform samples on the respective λ-regions. The “calibration runs” then use $s_{\lambda_{0}}=0.024$ for $\lambda_{0}=10^{-250}$, which we get from a large uniform sample on the whole state space, in Equation (11) and the resulting $s_{\lambda }$ in Equation (10). The “precision runs” use $\lambda_{1}=10^{-25}$ instead of $\lambda_{0}$ in Equation (11), with $s_{\lambda_{1}}≃10^{-24}$ obtained from the calibration runs, and $N_{\lambda }^{\prime }$ intermediate λ values. The graphs for the final calibration run and the first precision run, marked by ∗ in Figure 4 (bottom), are indistinguishable.

Regarding the computational effort, the comparison of the SCMC and the OTJ algorithms speaks strongly in favor of SCMC. The computation time scales roughly as $O\left(\left(N_{\lambda }+N_{\lambda }^{\prime }\right)N_{\tau }\sqrt{N_{s}}\right)$. Therefore, the final precision run takes about (2000+6000)50$\times \sqrt{10^{3}}/\left(200\sqrt{10^{6}}\right)≃10^{2}$ times longer than the direct production of a target sample by SCMC to evaluate $c_{\lambda }$, and the latter has higher accuracy.

In summary, we can produce samples uniformly distributed in λ-regions by an SCMC implementation of the OTJ method and so evaluate the content $c_{\lambda }$ as a function of λ. Our numerical results not only confirm that the OTJ method is viable; they also demonstrate that, for a sharply peaked target distribution, it is more efficient to evaluate the content from the target sample produced directly by SCMC than to use the more involved OTJ method. Further, we observe that the content estimate obtained by the OTJ method has a negative bias.

## 4. Conclusions

In conclusion, we presented an algorithmic study of the reliability and practical efficiency of the SCMC sampler for sampling quantum states through three explicit examples; yet another application of SCMC is reported in Reference [55]. In the first example, we produce samples of bound entangled states for dimensions 3×3, 3×4, 3×5, and 4×4. For some of these cases, no such samples were available before, and our method can also provide bound entangled states for higher dimensions. For example, we obtain nearly ten thousand two-qutrit bound entangled states in a few minutes through SCMC sampling, whereas several days of independent sampling yield 24 such states from $10^{10}$ candidates. We also observe that the 2×4 system is particular, as we could not find bound entangled states that obey the PPT-CCNR double criterion. Based on the numerical evidence, we conjecture that such states do not exist.

In the second example, we use the SCMC method for sampling quantum states in accordance with given target distributions for three-qubit and four-qubit systems. For the cases studied, SCMC is substantially more efficient than the alternatives we considered, and its cost appears to grow only moderately over the tested range as the dimension increases.

The third example is an SCMC implementation of the OTJ method for producing uniform samples on regions bounded by values of the target function (the λ-regions); the shortcomings of the original implementation are avoided. We find that, for the purpose of estimating the content of the λ-regions, direct SCMC sampling from the target distribution is much more efficient, and the values obtained by the OTJ method have a negative bias.

The SCMC sampler can be applied to many other sampling problems of quantum systems as long as the constraints can be described by inequalities. For example, one could produce samples of states that violate an inequality of the Bell kind. Multiple constraints can be efficiently applied in parallel. Moreover, due to the well-known channel–state duality, SCMC sampling can also be used to sample channels. We invite the readers to apply the method to their specific sampling problems.

## Figures and Tables

**Table 1:** Parameters used for the SCMC sampling (× → •) reported in Figure 1 and Figure 2: number $N_{\tau }$ of intermediate distributions, tolerance $a_{e}$ for the entanglement constraint, tolerance $a_{p}$ for the PPT constraint, yield of bound entangled states, and CPU time per distribution step.

System	$N_{\tau }$	$a_{e}$	$a_{p}$	Yield	Time
3×3	20	1,000	10,000	85%	11 s
3×4	2,000	1,000	10,000	70%	14 s
3×5	2,000	3,000	20,000	22%	15 s
4×4	2,000	3,000	500,000	40%	22 s
2×4	20	200	300	0%	7 s

**Figure A1:**
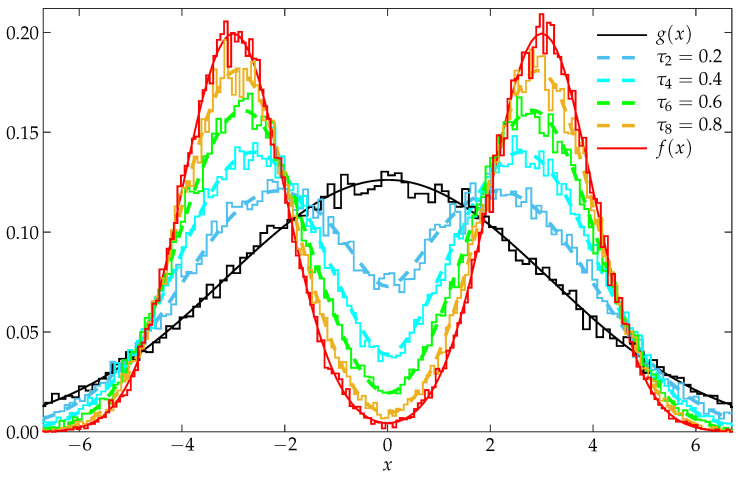
A simple illustration of SCMC in 1D. A single-peak reference sample is used to sample a target distribution with two peaks. The intermediate distributions are given by Equation (1) with $N_{\tau }=10$. Both the exact and the numerically estimated distribution for $10^{4}$ sample points are shown.

**Figure A2:**
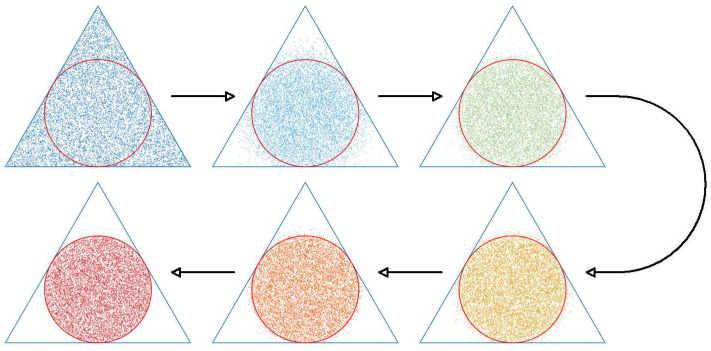
The physicality constraint is gradually imposed through $N_{\tau }=10$ intermediate distribution steps. The triangle represents the boundary of the probability simplex, while the circle represents the boundary of the physical region. The distributions are shown for the initial sample (uniform on the simplex, **top left**, $\tau_{0}=0$), for the final sample (uniform on the unit disk, **bottom left**, $\tau_{10}=1$), and for four intermediate samples ($\tau_{i}=i/10$ with i=2,4,6,8).

## Data Availability

The codes used for the numerical computations are available upon request.

## Abbreviations

    The following abbreviations are used in this article:

CCNR	Computational Cross Norm and Realignment
CPU	Central Processing Unit
ESS	Effective Sample Size
GB	Giga Byte
HMC	Hamiltonian Monte Carlo
PPT	Positive Partial Transpose
MC	Monte Carlo
MCMC	Markov Chain Monte Carlo
OTJ	Oh, Teo, and Jeong
RAM	Random-Access Memory
SCMC	Sequentially Constrained Monte Carlo
SMC	Sequential Monte Carlo

## Appendix. SCMC Algorithm

Generally speaking, one would implement SCMC as follows. First, try to generate a sample that is of considerable size. Pick the distribution step according to the scaling with the dimension *d* of the parameter space, i.e., $\propto d^{1.5}$ (see the last paragraph in Section 3.2). Then, construct the intermediate distributions according to the recipe in Equation (1). Parameterize the inequalities imposed on the target sample in accordance with Equation (2). By trial and error, adjust the Markov chain step size to reach an acceptance rate around 23.4%. Perhaps check the convergence by increasing the number of intermediate distributions; once convergence is ensured, optimize for efficiency by reducing the number of intermediate distributions.

The general algorithm takes in a set of $N_{s}$ initial sample points with distribution g(x) and finds a discretized sequence of density distributions $\left\{h_{i}\left(x\right)\right\}$ that “smoothly” bridges between g(x) and f(x). $\left\{h_{i}\left(x\right)\right\}$ takes into account both the change of the probability density distribution and the incorporation of the soft constraints given by Equation (1) to Equation (3).

In general, there can be $N_{\mathrm{MC}}$ MCMC iterations between each distribution step, and importance resampling is performed whenever the effective sample size (ESS) drops below a chosen threshold $N_{\mathrm{thres}}$. We employ the iteration kernel in the Metropolis algorithm, which results in sample points with updated weights for the *k*th iteration $w_{i}^{\left(k\right)}\propto \left[h_{i}\left(x_{i}^{\left(k\right)}\right)\right]/\left[h_{i-1}\left(x_{i}^{\left(k\right)}\right)\right]$. The ESS, that is ${\mathrm{ESS}}^{-1}=\sum_{k=1}^{N_{s}}{\left[w_{i}^{\left(k\right)}\right]}^{2}$, is a measure of sample degeneracy [56], i.e., a small ESS indicates that only a small portion of the sample points are filtered into the next step. This degeneracy can be greatly suppressed in SMC with finer distribution steps as compared with MCMC. In addition, the SMC algorithms can be conveniently implemented with parallel computation for speed-up. The general outline of our SCMC algorithm is given on the next page.

**SCMC** Sequentially constrained Monte Carlo algorithm
1: Generate an initial sample $\left\{\rho^{\left(1:N_{s}\right)}\right\}$ with distribution g(ρ) 2: Assign weight $w_{0}^{\left(k\right)}=1/N_{s}$ for $k=1:N_{s}$ 3: **for** $i=1:N_{\tau }$**do** 4: Evaluate $h_{i}\left(\rho^{\left(1:N_{s}\right)}\right)$ with indicators $I_{\kappa ,i}\left(\rho^{\left(1:N_{s}\right)}\right)$ 5: **for** $j=1:N_{\mathrm{MC}}$ **do** 6: **if** j=1 **then** 7: Update $w_{i}^{\left(k\right)}=w_{i-1}^{\left(k\right)}\frac{h_{i}\left(\rho^{\left(k\right)}\right)}{h_{i-1}\left(\rho^{\left(k\right)}\right)}$ for $k=1:N_{s}$ 8: Normalize $w_{i}^{\left(k\right)}$ 9: Calculate $\mathrm{ESS}={\left[\sum_{k=1}^{N_{s}}{\left(w_{i}^{\left(k\right)}\right)}^{2}\right]}^{-1}$ 10: **if** $\mathrm{ESS}<N_{\mathrm{thres}}$ **then** 11: Importance resampling according to $w_{i}^{\left(1:N_{s}\right)}$ 12: Reset $w_{i}^{\left(1:N_{s}\right)}=1/N_{s}$ 13: **end if** 14: **end if** 15: Propagate $\left\{\rho^{\left(1:N_{s}\right)}\right\}$ with MCMC transition kernels 16: Accept/reject the new points 17: **end for** 18: **end for**

Figure A1 illustrates a simple one-dimensional example of SMC. The target distribution f(x) has two peaks, and we sample it by starting with random samples drawn from a single-peak normal distribution g(x). A simple example that illustrates how the physicality constraint can be effectively imposed is shown in Figure A2. The reference sample is uniform in the probability simplex of the distorted trine measurement of Reference [54] on a single-qubit system and it is filtered through $N_{\tau }=10$ intermediate constrained uniform distributions, thereby imposing the physicality constraint by taking κ(ρ) to be the smallest eigenvalue of the density matrix of state ρ.

The computational efficiency of the SCMC algorithms depends on a set of parameters—the number of reference sample points $N_{s}$, the number of distribution steps $N_{\tau }$, the number of Markov chain iterations $N_{\mathrm{MC}}$ that the entire sample is advanced by a kernel of choice in each distribution step (see below) and the scaling parameter *a*—which need to be calibrated for the sampling problem at hand. For most of the examples presented in this paper, we fix $N_{\mathrm{MC}}=15$ and adjust other parameters in accordance with the criterion set out in Reference [57].

Generally, the number of Markov chain iterations, $N_{\mathrm{MC}}$, is chosen according to [57], $N_{R}=\left\lceil \log_{1-ar}\left(q\right)\right\rceil$, where $N_{R}$ is the required number of Markov chain steps such that there is a (1−q) probability that each point is moved at least once during the Markov chain procedure, which has an acceptance rate of ar. To put in some numbers for illustration, $N_{\mathrm{MC}}=7$ when q=0.01 and ar=0.25 for a typical Metropolis–Hastings Markov chain, which has a most efficient acceptance rate of 0.234 [58], and we choose $N_{\mathrm{MC}}=15$ for convenience throughout the paper.

Regarding the choice of kernel, the most standard Gaussian kernel is an option. The kernel we are using here is the “adaptive Metropolis kernel” of Section 2 in Reference [59], which has its origin in Reference [60].

To reduce the sample degeneracy without compromising its efficiency by too much, we set the threshold of ESS at $N_{\mathrm{thres}}=\frac{4}{5}N_{s}$ and importance resampling is executed once ESS falls below $N_{\mathrm{thres}}$. The sample correlation resulting from importance resampling is attended to by the Markov chain iterations that follow. To reduce the correlation from the final importance resampling before the SCMC algorithm ends, we filter the system through 20 additional Markov chain iterations.
